# Energy communities: engaging people and technologies in the future of energy

**DOI:** 10.12688/openreseurope.15242.1

**Published:** 2022-12-12

**Authors:** Tatiana Loureiro, Paula Jiménez Argumosa, Aggeliki Aggeli, Marta Arniani, Julia Blanke, Blanca Barrios

**Affiliations:** 1 R2M Solution Spain SL, Madrid, Spain; 2 TRAZA Territorio, Madrid, Spain; 3 Aalborg University Copenhagen, Copenhagen,, Denmark; 4 Futuribile, Paris, France; 5 Munster Technological University,, Cork, Ireland

**Keywords:** Citizens; energy communities; people; technology; energy.

## Abstract

This is a summary of the clustering workshop at the Sustainable Places 2022 conference. The H2020 funded projects LIGHTNESS, HESTIA, LocalRES and CREATORS came together to shed light on the importance of engaging people and unlocking the potential of technologies to promote energy communities and meet the EU’s ambition of a joint net-zero emission of greenhouse gases by the year 2050. To ensure a people-centric, sustainable, just, and innovative energy future, citizens and experts must be brought into dialogue to co-design new ways of organising around energy.

## Disclaimer

The views expressed in this article are those of the author(s). Publication in Open Research Europe does not imply endorsement of the European Commission.

## Introduction

Four EU funded projects, that are working on the development of innovative technical solutions for the stimulation of energy communities, have come together in a clustering workshop within the conference Sustainable Places 2022 (September 8, in Nice, France) to discuss the challenges of engaging citizens into the co-design of said communities alongside technical experts, in order to ensure a just energy transition.

The objective of this workshop is to shed light on the importance of engaging people and unlocking the potential of technologies to promote energy communities, meeting the EU’s ambition of a joint net-zero emission of greenhouse gases by the year 2050. To ensure a people-centric, sustainable, just, and innovative energy future, citizens and experts must be brought into dialogue to co-design new ways of organisation around energy.

These four projects are working on different typologies of energy communities, and their approach to these challenges is varied. Instead of a techno-centric approach, these projects presented their methods to engage people and co-design technologies to forward a clean and fair energy transition.

## LIGHTNESS: bridging social and technological innovation strategies

The LIGHTNESS (market uptake of citizen energy communities enabLing a hIGH peneTratioN of renewable Energy SourceS) project aims to shift the energy culture and pave the way towards a just energy transition by supporting citizen energy communities (CECs) through social engagement, regulatory roadmaps, a low-cost technological package, and innovative business models. One of the significant yet challenging goals of the project, and of energy communities at large, is to generate long-lasting changes in the way we relate to energy, to make it more just and sustainable. This not only takes technological innovation, but also complex engagement processes that manage to involve diverse people and change their day-to-day practices, perceptions, and knowledge about energy.

Involving people in the energy transition is complex and takes creative social innovation approaches that intertwine with technological ones. In accordance, within LIGHTNESS, three key pillars have been envisioned to devise strategies. The pillar of exploration is about revealing the needs, wants and perceptions of participants in relation to energy. Recruitment gives insight into how a value proposition of the potential energy community can spark the interest of end-users’ of energy, and co-design discusses how a participatory process can engage participants in collaboratively proposing key aspects of the community such as organizational models, technological tools, etc. Active engagement of a diverse and representative group is crucial, and implicates generation and gender specific tools for elderly, younger, and female end-users, as well as lower income households, illiterate, businesses, and institutional actors. Methodologies from social sciences and humanities, such as interviews and questionnaires, as well as facilitation tools, have shown great results. For instance, in adjusting engagement plans to people’s concerns, and the codesign of playful and gamified features of the app to help end-users of energy satisfy their needs (i.e. getting daily information about ways to change their practices to be more energy efficient).

### Housing community of apartment blocks in Wroclaw (Poland)

In the largest city of Poland, Wroclaw, two apartments are piloting energy communities. Photovoltaic panels are already installed on the roof and directed towards energy consumption in common areas. Within the project, the energy community will scale up, for which the role of the building manager is fundamental as they have important knowledge about the building and residents.

The biggest challenge in this pilot has been recruitment, given that most residents are elderly and immobile. The insider knowledge of the building manager, revealed through an in-depth interview, has been key to test different strategies and finally, through door-to-door visits, be able to start engaging end-users. This experience shows that social innovation is key and was relatively ignored at first. Besides, the pilot leader encountered difficulties in trying to explain the concept of the energy community to the residents, for which discursive creativity is needed, to connect with inner needs and adjust while slightly increasing the energy literacy of residents.

### Residential communities (Netherlands)

The Dutch case study combines two pilot sites in different locations: Woerden and Quatre Bras. The pilot in Woerden consists of social housing and is composed of ZOM (zero-on-meter) houses, that went through a net-zero retrofitting by the construction company BAM. The second pilot is the new urban development Quatre Bras, which consists of single-family houses of a wealthier population.

In the Woerden pilot, door-to-door visits showed the limited energy and technological literacy of residents, which led to building the common language narratives to explain energy sharing and how citizens can get involved in the energy system. Time is, in this case, key to generating the tacit knowledge necessary to change perceptions, behaviours and practices around energy, by using the app but also a collective governance structure and learning by sharing experiences. A community game allowed breaking of the ice among end-users and show their inner motivations to engage in an energy community, which seem to be economic savings and comfort. This is being used to design the app and engagement process accordingly.

In the Quatre Bras pilot, *st*reet interviews conducted showed that people are quite energy and technologically literate but are mostly constrained through their time budget. Meanwhile, given their work and caring responsibilities, most end-users cannot actively engage, for whom different levels of participation are more suitable. In this quite typical case, the consolidation of a reduced leading group could work to move the community forward and socialize key milestones or learnings with the rest.

### Electric cooperative in Alginet, Valencia (Spain)

Alginet is a village of 13,000 inhabitants located 25 km from Valencia, in eastern Spain. Alginet’s electric cooperative was created in 1930 by a group of citizens, due to the need for electricity supply in the town and the lack of interest from the electric companies to electrify small population centres. Today the cooperative has almost 6,000 users. Besides the commercialization and distribution of electric energy, the cooperative also plays a major social role in the town by investing and redistributing benefits among the end users, mitigating energy poverty, and supporting climate change adaptation.

It already operates as an energy community; however, the cooperative looks to empower and better inform participants about energy consumption and ways to upscale the renewable generation. Thus, the app will be a valuable asset for end-users to track their consumption, however, in person participatory workshops to consolidate an active group that shares learnings and roadmaps a path to increase the photovoltaic shared consumption is key. An important challenge is getting people motivated to codesign, without many references of how this community could operate. It takes changing the energy culture, from passive to active users, which takes time and encounters to approach concerns, worries, and needs.

### Smart Condo in Cagliari (Italy)

In Italy, one apartment building or ‘condominium’ with eight apartments in the village of Cagliari, Sardinia, is involved. With financial aid from the “SuperEcoBonus 110 %” subsidy the building is being renovated and equipped with smart meters. Residents have strong community ties, and organize friendly gatherings between various households, and more formal meetings to discuss issues regarding the condominium. Regarding the creation of an energy community, they have been learning and discussing about the photovoltaic installation, the batteries, and the app. The existing social ties between residents have enriched the social and technological innovation, promoting a smooth transmission of knowledge from the more energy literate to those with less knowledge. A compelling aspect of the community is the robust sense of community and potential of relationships, for all participants to apprehend and slowly shift the energy culture of the condo.

All in all, the experience with the pilot sites of the LIGHTNESS project shows the interdependency of technological and social innovation, and thus, the need to develop tools to bridge these crucial areas. A driving challenge when promoting energy communities in a just environmental framework is approaching the sociocultural dimension of the energy transition. Paving the way for end-users of energy to learn, make informed decisions, adapt their daily practices, and take on a community-led initiative takes, especially, innovative social methods. In this sense, Social Sciences and Humanities count with important insights to research, propose and implement ways of forwarding a new energy culture.

## HESTIA: an inclusive and participatory engagement process for developing a demand response (DR) platform

HESTIA (Holistic dEmand response Services for European residenTIAl communities) is a Horizon 2020 project which aims to develop a cost-effective next generation DR platform by encouraging people to engage in flexibility sharing and grid balancing. There are three important starting points for the project’s engagement strategy: first, understanding demand response at home requires an appreciation of the underlying rhythms and dynamics of everyday life. Second, that the technical, quantitative understanding of energy, developed from building professionals, is considerably different from the experiential, qualitative understanding of consumers, such as householders. Third, that the sharing of expertise and the understanding of the expectations of both consumers and technical experts needs to be equally considered when designing DR solutions. At the core of the project’s engagement is co-creation, as a way to involve householders, researchers and other community stakeholders, as well as for providing a platform within the consortium for negotiating and validating the project’s pathways. Co-creation is being performed through face-to-face and virtual workshops and household interactions. Face-to-face workshops always include visual and interactive methods for engagement and are tailored to each pilot energy and cultural context.

Preliminary observations from the pilot sites suggest that introducing energy technologies and systems into homes is not as straightforward as technology developers often conceptualise. The lived experiences of householders show that everyday life practices in the home need to be well coordinated with the new technologies in order to ensure a successful introduction of energy flexibility. Moreover, interactions with citizens display different degrees of confidence with digital means: we expect that the digital literacy of participants will have a large influence on the mid- and long-term adoption of new flexibility interfaces, regardless of the technology readiness level and technical efficiency of the systems deployed. Through these initial findings, the project has developed recommendations for the engagement of different household typologies (e.g. families with children, intergenerational households, and retirees) in the process of developing the DR platform and recommendations for the design and technical development of the platform, such as suggestions for the frequency of interactions through the platform as well as recommendations for ensuring a diverse inclusion and accessibility in the platform. Furthermore, recommendations have been written on how to engage the whole community, rather than individual users, and how this can contribute to the strengthening of the newly formed energy communities in the pilot sites. 

Furthermore, a promising avenue for work which emerged through the ongoing participatory interactions is the gender dynamics at home and the influence they have on the effectiveness of flexibility measures: indeed, gender dynamics shape the coordination of everyday housekeeping with the newly introduced digital housekeeping of energy technologies. There is currently a miscoordination between the everyday housekeeping practices and the technology-related digital housekeeping practices. Initial findings show that the uneven distribution of labour between men and women which characterises many aspects of society can be found also in the use and management of energy at home, particularly regarding the operation and control of energy technologies. Experiences of trust and control are gendered too, suggesting that engagement activities should pay special attention to include all participants in the understanding of the process and of the equipment deployed. Through targeted focus groups, e.g. women-only workshops in our Italian and Dutch pilot sites, we further explore the issues around the gendering of the technologies entering the homes, as well as how to better support householders, particularly women, in the uptake and domestication of these new technologies. We expect such engagement activities to empower women participants, improve the effectiveness of the project and promote equality within the households.

Four themes emerge as key for securing citizen engagement in the project for the mid-to-long term: the consideration of the coordination and gendering of household labour; addressing the digital literacy and skills of citizens in the pilot; the frequency of interactions, which needs to be balanced; and the need for frequent reviewing of the motivation of citizens, without taking for granted that their initial reasons to engage will stay the same over the project lifetime. Regarding technology interfaces, we identify four areas of work: personalisation; a regular feedback loop between the users and the platform; a careful consideration of the frequency of notifications; and overall support, considering the different degrees of digital literacy.

In conclusion, the findings in HESTIA so far, point to a need for appropriate ways to understand and interpret householders’ gendered know-how of everyday life practices in order to better customise the DR platform, considering local social norms and everyday conventions.

## LocalRES: A case study of European small-town renewable energy communities (RECs)

The main objective of the LocalRes (Empowering local renewable energy communities for the decarbonisation of the energy systems) project is to empower local renewable energy communities for the decarbonisation of the energy systems. Currently there is a shift of paradigm in the energy sector from a purely centralised to a more decentralised approach and from mainly passive to active prosumers.

Three main social dimensions of relevance can be identified in the energy transition: First, the energy transition needs people to accept new technologies in their daily lives and adapt their behaviour accordingly. Second, people must engage on an individual and on a community level. Finally, they need to decide on investments and participate in the operation of the assets and systems. Social innovation along all three dimensions is necessary.

However, a recurring issue when trying to implement new technical solutions is that people need to be kept on board. RECs are a vehicle to address some of the social challenges of the energy transition.

LocalRES aims to develop supporting tools, like an app or webpage, to help renewable energy communities with building and selecting community goals and scenarios, to understand technical, economic, environmental, and social implications as well as providing a platform for communication and community building. If the involvement of citizens is taken serious in the energy transition, it needs to be made sure that relevant technologies are developed with them instead of just for them.

This approach is called participatory design or co-design and the idea is to involve all relevant stakeholders in the development process from a very early stage. It gives everyone a voice and provides valuable insights not only for the products to be developed but also into the community itself.

In LocalRes a two-step approach was used to understand the local energy communities in the project pilot sites. A first series of workshops was held with representatives together with the community leaders to identify specific goals and scenarios that are considered to be relevant for the different communities. Based on this initial understanding a second phase of workshops was developed, which ran with the citizens of the communities to collect their feedback on these goals and scenarios, but also on their needs and concerns around their own energy community. Both qualitative and quantitative data were collected.

The LocalRes pilot sites are all small towns in different regions across Europe (Berchidda, Italy, Ollersdorf, Austria, Ispaster, Spain, Kökar, Finland). Every community was different in its stage of development and focused on different goals and scenarios. Quantitative analysis showed that the target platforms need to consider different demographics, technological preferences and literacies.

The qualitative feedback from participants during the citizen workshops revealed three main areas of interests: technical, economic, and social. However, one of the main focuses was that the development of the community itself was a topic of primary importance to many participants. Communication and coordination between members of the community was mentioned frequently as an important aspect of supporting activities and tools.

A limitation of the study has been the selection bias. Only interested parties took part in the workshops and a strong overrepresentation of older, male participants could be observed.

It can be concluded that RECs are a novel approach to address the energy transition. A particular focus needs to be at the social and not just at the technical dimension. Co-creation can foster active engagement and empower citizens to take part and make informed decisions regarding energy community relevant topics.

## CREATORS: Finding the “key person” as local initiator of the energy community

CREATORS (CREATing cOmmunity eneRgy Systems) aims to accelerate the integration of community energy systems (CES) across Europe by supporting local initiators and local service providers throughout the entire life cycle of a project.

Within CREATORS we are developing and advancing a set of applications and services that will help in the initiation, planning, implementation and operation of the CES.

To ensure a high market uptake of the CREATORS’ tools and services, we are also designing viable business models built upon verifiable data on technical and financial performance as well as contracting protocols. Additionally, we will define and commercialise “CES-as-a-Service”, a fully integrated solution for energy communities.

Even though CES are generating a lot of interest, they are still a new model and require not only a strong technological package, but also the creation of a bridge between technology and the participants of these kinds of projects.

From the technology perspective, in the CREATORS project we are developing applications such as a simulation and emulation engine (SEE), digital twins and a management and trading platform for energy communities. These technologies and advancements are being tested in several pilot projects across Europe, so far in more than ten different sites and by the end of the CREATORS project, we will be supporting nearly 24 communities. The solutions of CREATORS for energy communities will have a big replication potential.

The CREATORS pilot projects are very different, covering big consumers such as business parks, apartment buildings, industrial consumers, tertiary sector, public buildings, etc. When initiating a new CES project, CREATORS identified that it is crucial to find a key person that will serve as a link between the service provider CREATORS and the final users of the community.

This key person, usually a local initiator or a local service provider is essential in a project because will understand the needs of the people forming the community and work together with the service provider towards a viable energy community onsite. The CREATORS team performs simulations and emulations, through a digital twin technology, until the optimal configuration of the energy community is found, both regarding technical and economic aspects.

This key person also facilitates the interaction with the final users, providing trust and sometimes even providing explanations in the local language. It is also very important to organise workshops with site owners and final users, communicating about the importance of energy communities and the social, environmental, and economic benefits that come along. Providing information in the local language will help in the engagement and allow the members of the community to participate in the decision-making process.

The experience with the pilot sites of CREATORS shows that it is challenging to involve final users in an energy community, especially when the projects are big in size and involve many different users. It is crucial to find a key person, a local initiator, that will be the bridge between the technology provider and the members of the community, and support in the decision making considering the local needs of the community. Moreover, this person can also overcome barriers related to local language and create trust.

## Conclusions

The presentation of the approaches, methodologies, solutions, and experiences being tested in different types of energy communities within these projects led to a high-level discussion. LIGHTNESS illustrated how an environmental justice framework is being implemented to co-create energy communities through technological and social innovation. HESTIA demonstrated how engagement and gender inclusion, paired with energy and digital literacy, can boost the effectiveness of demand response technologies. LocalRES drove reflections about the role of local representatives and citizens in co-designing technologies and energy communities. CREATORS showed how stakeholder needs in industrial sites are met using technological solutions.

The following conclusions were drawn from the debate:

- The democratization of energy can only be achieved if citizens are brought as active participants and not mere consumers, making sure to ensure a people-centric approach through all steps, even from the design of technological solutions.
- Understanding the needs, the wants and the habits of consumers is a fundamental step in order to develop energy communities.
- Each community has their own characteristics and problems; therefore, a personalized approach is necessary to create technological solutions. Consequently, it is difficult to have a standard methodology, but good practices can be established and replicated.
- Bridging the gap between people and technology is mandatory to achieve a just and innovative energy future, and citizen energy communities are an excellent solution to help achieving the EU’s decarbonization objectives.

## Data Availability

No data are associated with this article
