# Supplementary material for: Energy communities: engaging people and technologies in the future of energy
Source: Open Res Eur. 2022 Dec 12;2:137. [Version 1] doi: 10.12688/openreseurope.15242.1 (PMC10445896; doi:10.12688/openreseurope.15242.1)
Supplement: Peer review history

# Peer review: Reviewer response for version 1

**DOI:** 10.21956/openreseurope.16484.r30481

**Published:** 2023-01-10

**Authors:** Tineke van der Schoor

**Affiliations:** 1 Research Centre for Built Environment NoorderRuimte, Hanze University of Applied Sciences, Groningen, The Netherlands

- The topic of energy communities is only very briefly described. In a full article this should be elaborated upon, as it stands now, the introduction is not sufficient. Also, no references to the extensive literature on the subject are provided. 
- Differing views and opinions are not discussed. The article consists of a description of cases, which are really very interesting, but discussion of findings and evaluating findings in the light of the literature is lacking.
- Theoretical approaches are only mentioned, not presented. Please reflect on these approaches in more detail and come back to them in the conclusions.
- The descriptions of the projects are clear, but there are no references to back up the statements. Therefore, I advise to include more information about the methodology in the different projects. How many interviews, survey respondents, or focus groups were carried out? How was the analysis done?
- The conclusions at the end of the article appear rather suddenly, this should be expanded to a at least a subsection for each conclusion. Here you can draw comparisons between the four EU-projects and explain commonalities, differences and new connections. Also, how was the workshop set up in which you came to these conclusions?
- I enclose a working paper and a brief article on my literature review on community energy, I hope this will be useful for you ^1^.

Where applicable, are recommendations and next steps explained clearly for others to follow? (Please consider whether others in the research community would be able to implement guidelines or recommendations and/or constructively engage in the debate)

Partly

Does the article adequately reference differing views and opinions?

No

Are all factual statements correct, and are statements and arguments made adequately supported by citations?

Partly

Is the rationale for the Open Letter provided in sufficient detail? (Please consider whether existing challenges in the field are outlined clearly and whether the purpose of the letter is explained)

Partly

Is the Open Letter written in accessible language? (Please consider whether all subject-specific terms, concepts and abbreviations are explained)

Yes

Reviewer Expertise:

community energy, heritage, social sciences

I confirm that I have read this submission and believe that I have an appropriate level of expertise to confirm that it is of an acceptable scientific standard, however I have significant reservations, as outlined above.

## References

[1] : The power of friends and neighbors: a review of community energy research. *Current Opinion in Environmental Sustainability* .2019;39: 10.1016/j.cosust.2019.08.004 71-80 10.1016/j.cosust.2019.08.004

# Peer review: Reviewer response for version 1

**DOI:** 10.21956/openreseurope.16484.r30482

**Published:** 2023-01-05

**Authors:** Richard Bull

**Affiliations:** 1 School of Architecture, Design and the Built Environment, Nottingham Trent University, Nottingham, UK

This is an interesting overview of an important topic. The four cases are useful and the discussion section at the end reflects on the purpose of them. This would have been useful at the start as it wasn't clear to me, from the introduction section whether this was a review of outputs from the conference or the four cases - the language is a bit muddy. 

 I appreciate that time is space in articles like this but a lot of assertions are made regarding what energy communities are alongside the benefits and challenges of engagement are but these are never spelt out or defined. Maybe there's an issue with the formatting of the version I've got but there are no references at all to support the any of the arguments made. I've written extensively on this and provide some of the recent works I've used or contributed  - see below and attached ^1^.

 So in short - the 4 cases studies are interesting and presented clearly but due to a lack of referencing or academic reflection much of it feels like assertion. For example, in the intro, para 3 - why must people be brought into dialogue; section 2 para 2 - what is the value proposition'  - the final para of this section that mentions 'social science and humanities' is not clear also.

 Section 2 - para 2 - what are the recommendations for engaging a whole community?

 The observation on gender dynamics is important but again, there is no detail - such as?

 I hope these observations make for an improved version.

 Suggested Refs. 

 Ambrose, A. (2020) Walking with Energy: Challenging energy invisibility and connecting citizens with energy futures through participatory research. Futures, 117. 

 Arnstein, S. (1969). "A Ladder of Citizen Participation." Journal of the American Institute of Planners 35: 216-224. 

 Bull R., and Eadson, W. (2020) Citizen engagement in local energy decision-making: literature and policy background. https://www.shu.ac.uk/centre-regional-economic-social-research/publications/citizen-engagement-in-local-energy-decision-making-literature-and-policy-background 

 Bull, R., and Janda, K. (2018). Beyond feedback: introducing the 'participation gap' in organisational energy management. Building Research and Information 46 (3), pp. 200-215. 

 Bull, R., Lemon, M., Everitt, D., and Stuart, G. (2015). Moving beyond feedback: Energy behaviour and local engagement in the United Kingdom. Energy Research and Social Science 8 32-40. 

 Bull, R., J. Petts, et al. (2008). "Social Learning from Public Engagement: Dreaming the impossible?" Journal of Environmental Management and Planning 51(5): 703-718. 

From discovering to delivering: A critical reflection on eco-feedback, application design, and participatory research in the United Kingdom

 ML Chalal, B Medjdoub, R Bull, R Shrahily, N Bezai… - Energy Research & Social Science, 2020

 Chilvers, J. and Longhurst, N. (2016) Participation in Transition(s): Reconceiving Public Engagements in Energy Transitions as Co-Produced, Emergent and Diverse, Journal of Environmental Policy & Planning, 18:5, 585-607, DOI: 10.1080/1523908X.2015.1110483 

 Eadson, W., and van Veelen, B. (2021) Assemblage-democracy: Reconceptualising democracy through material resource governance. Political Geography 88 https://doi.org/10.1016/j.polgeo.2021.102403.  

 Gustafsson, S, Ivner, J and Palm, J (2015) Management and stakeholder participation in local strategic energy planning: Examples from Sweden. Journal of Cleaner Production 98 pp. 205-212.  

 Sovacool, B, (2014) What are we doing here? Analyzing fifteen years of energy scholarship and proposing a social science research agenda. Energy Research & Social Science 1, 1-29.

Where applicable, are recommendations and next steps explained clearly for others to follow? (Please consider whether others in the research community would be able to implement guidelines or recommendations and/or constructively engage in the debate)

Yes

Does the article adequately reference differing views and opinions?

Partly

Are all factual statements correct, and are statements and arguments made adequately supported by citations?

No

Is the rationale for the Open Letter provided in sufficient detail? (Please consider whether existing challenges in the field are outlined clearly and whether the purpose of the letter is explained)

Partly

Is the Open Letter written in accessible language? (Please consider whether all subject-specific terms, concepts and abbreviations are explained)

Yes

Reviewer Expertise:

Public engagement, energy behaviour change, feedback and ICT tools

I confirm that I have read this submission and believe that I have an appropriate level of expertise to confirm that it is of an acceptable scientific standard, however I have significant reservations, as outlined above.

## References

[1] : Hot air or new energy: Are we seeing signs of improved citizen engagement in district heating schemes?. *Science Talks* .2022;4: 10.1016/j.sctalk.2022.100096 10.1016/j.sctalk.2022.100096

[2] : Walking with Energy: Challenging energy invisibility and connecting citizens with energy futures through participatory research. *Futures* .2020;117: 10.1016/j.futures.2020.102528 10.1016/j.futures.2020.102528

[3] : A Ladder Of Citizen Participation. *Journal of the American Institute of Planners* .1969;35(4) : 10.1080/01944366908977225 216-224 10.1080/01944366908977225

[4] : Citizen engagement in local energy decision-making: literature and policy background.2020; 10.7190/cresr.2020.8937358309 10.7190/cresr.2020.8937358309

[5] : Beyond feedback: introducing the ‘engagement gap’ in organizational energy management. *Building Research & Information* .2018;46(3) : 10.1080/09613218.2017.1366748 300-315 10.1080/09613218.2017.1366748

[6] : Moving beyond feedback: Energy behaviour and local engagement in the United Kingdom. *Energy Research & Social Science* .2015;8: 10.1016/j.erss.2015.04.006 32-40 10.1016/j.erss.2015.04.006

[7] : Social learning from public engagement: dreaming the impossible?. *Journal of Environmental Planning and Management* .2008;51(5) : 10.1080/09640560802208140 701-716 10.1080/09640560802208140

[8] : From discovering to delivering: A critical reflection on eco-feedback, application design, and participatory research in the United Kingdom. *Energy Research & Social Science* .2020;68: 10.1016/j.erss.2020.101535 10.1016/j.erss.2020.101535

[9] : Participation in Transition(s): Reconceiving Public Engagements in Energy Transitions as Co-Produced, Emergent and Diverse. *Journal of Environmental Policy & Planning* .2016;18(5) : 10.1080/1523908X.2015.1110483 585-607 10.1080/1523908X.2015.1110483

[10] : Assemblage-democracy: Reconceptualising democracy through material resource governance. *Political Geography* .2021;88: 10.1016/j.polgeo.2021.102403 10.1016/j.polgeo.2021.102403

[11] : Management and stakeholder participation in local strategic energy planning – Examples from Sweden. *Journal of Cleaner Production* .2015;98: 10.1016/j.jclepro.2014.08.014 205-212 10.1016/j.jclepro.2014.08.014

[12] : What are we doing here? Analyzing fifteen years of energy scholarship and proposing a social science research agenda. *Energy Research & Social Science* .2014;1: 10.1016/j.erss.2014.02.003 1-29 10.1016/j.erss.2014.02.003

# Peer review: Reviewer response for version 1

**DOI:** 10.21956/openreseurope.16484.r30485

**Published:** 2023-01-05

**Authors:** Mirna Gržanić

**Affiliations:** 1 Faculty of Electrical Engineering and Computing, University of Zagreb, Zagreb, Croatia

The Open Letter describes following projects LIGHTNESS, HESTIA, LocalRES and CREATORS related to citizens engagement in energy communities and demand response presented on conference Sustainable Places 2022. The Letter describes pilot projects developed in each project in detail highlighting the main benefits achieved with pilots from social, environmental, technological and financial point of view.

 This Letter is very useful paper to address the main issues and achievements in the citizens related EU projects. 

 As it is not a research or a review paper, the reviewer cannot give any comment regarding the methodology, model, novelty or case studies and results (except Warsaw is the largest city in Poland, not Wroclaw. )

Where applicable, are recommendations and next steps explained clearly for others to follow? (Please consider whether others in the research community would be able to implement guidelines or recommendations and/or constructively engage in the debate)

Not applicable

Does the article adequately reference differing views and opinions?

Yes

Are all factual statements correct, and are statements and arguments made adequately supported by citations?

Partly

Is the rationale for the Open Letter provided in sufficient detail? (Please consider whether existing challenges in the field are outlined clearly and whether the purpose of the letter is explained)

Yes

Is the Open Letter written in accessible language? (Please consider whether all subject-specific terms, concepts and abbreviations are explained)

Yes

Reviewer Expertise:

Energy communities, demand response, the role of final customer in clean energy transition

I confirm that I have read this submission and believe that I have an appropriate level of expertise to confirm that it is of an acceptable scientific standard.
